# Graves disease and metastatic hormonal-active Hürthle cell thyroid cancer: A case report

**DOI:** 10.1097/MD.0000000000026384

**Published:** 2021-06-25

**Authors:** Nikola Besic, Barbara Vidergar-Kralj, Katja Zaletel, Cvetka Grasic-Kuhar

**Affiliations:** a Department of Surgical Oncology, Institute of Oncology Ljubljana; b Faculty of Medicine Ljubljana; c Department of Nuclear Medicine, Institute of Oncology Ljubljana; d Department of Nuclear Medicine, University Clinical Center Ljubljana; e Department of Medical Oncology, Institute of Oncology Ljubljana, Zaloška 2, Ljubljana, Slovenia.

**Keywords:** case report, Graves disease, hyperthyroidism, metastatic Hürthle cell thyroid cancer, treatment

## Abstract

**Rationale:**

A hormone-active metastatic Hürthle cell thyroid carcinoma (HCTC) and Graves disease (GD) present a therapeutic challenge and is rarely reported.

**Patient concerns:**

We present a 64-year-old male patient, who had dyspnea and left hip pain lasting 4 months. He had clinical signs of hyperthyroidism and a tumor measuring 9 cm in diameter of the left thyroid lobe, metastatic neck lymph node and metastases in the lungs, mediastinum, and bones.

**Diagnosis:**

Laboratory findings confirmed hyperthyroidism and GD. Fine-needle aspiration biopsy and cytological investigation revealed metastases of HCTC in the skull and in the 8th right rib. A CT examination showed a thyroid tumor, metastatic neck lymph node, metastases in the lungs, mediastinum and in the 8th right rib measuring 20 × 5.6 × 4.5 cm, in the left acetabulum measuring 9 × 9 × 3 cm and parietooccipitally in the skull measuring 5 × 4 × 2 cm. Histology after total thyroidectomy and resection of the 8th right rib confirmed metastatic HCTC.

**Interventions:**

The region of the left hip had been irradiated with concomitant doxorubicin 20 mg once weekly. When hyperthyroidism was controlled with thiamazole, a total thyroidectomy was performed. Persistent T3 hyperthyroidism, most likely caused by TSH-R-stimulated T3 production in large metastasis in the 8th right rib, was eliminated by rib resection. Thereafter, the patient was treated with 3 radioactive iodine-131 (RAI) therapies (cumulative dose of 515 mCi). Unfortunately, the tumor rapidly progressed after treatment with RAI and progressed 10 months after therapy with sorafenib.

**Outcomes:**

Despite treatment, the disease rapidly progressed and patient died due to distant metastases. He survived for 28 months from diagnosis.

**Lessons:**

Simultaneous hormone-active HCTC and GD is extremely rare and prognosis is dismal. Concomitant external beam radiotherapy and doxorubicin chemotherapy, followed by RAI therapy, prevented the growth of a large metastasis in the left hip in our patient. However, a large metastasis in the 8th right rib presented an unresolved problem. Treatment with rib resection and RAI did not prevent tumor recurrence. External beam radiotherapy and sorafenib treatment failed to prevent tumor growth.

## 1 Intruduction

Thyroid-stimulating hormone (TSH) is the leading physiological stimulator of the growth and function of thyrocytes, acting through the TSH receptor (TSH-R).^[1]^ Graves disease (GD) is an autoimmune disorder characterized by diffuse follicular cell hyperplasia and excessive production of thyroid hormones which is stimulated by antibodies against TSH-R (anti-TSH-R) that mimic the action of TSH.^[2,3]^ High serum anti-TSH-R are reported to stimulate the growth of thyroid cancer and metastases.^[4,5]^ However, in patients with thyroid cancer, the prevalence of GD is very low and the present guidelines do not give specific recommendations for the treatment of patients with differentiated thyroid cancer who have GD.^[6]^

Hürthle cell thyroid carcinoma (HCTC) is a rare type of thyroid carcinoma ^[7]^ which accounts for around 3% of all thyroid malignancies.^[8]^ The presence of distant metastases is an independent prognostic factor for disease-specific survival in patients with HTCT.^[9]^ In one of our recent publications,^[10]^ we reported our experience with the treatment of 32 patients with metastatic HCTC who were treated by surgery, radioiodine therapy, external beam irradiation, and chemotherapy in our tertiary center from 1972 to 2011. There are not any reports on a hormone-active metastatic Hürthle cell thyroid carcinoma and Graves disease in the literature. In the present case report, we describe the progressive course of the disease and management of a patient with Graves disease and metastatic HCTC who was treated by external beam irradiation with concomitant chemotherapy, surgery, radioiodine therapy, and targeted therapy.

## 2 Case presentation

A 64-year-old male patient, a retired married truck driver, who had chronic obstructive pulmonary disease, type II diabetes, and paroxysmal atrial fibrillation presented with dyspnea and left hip pain lasting 4 months before the first examination at the Institute of Oncology Ljubljana in November 2017. At diagnosis, he had clinical signs of hyperthyroidism and a tumor measuring 9 cm in diameter of the left thyroid lobe which was partially fixed against the trachea and mediastinum. Laboratory findings confirmed hyperthyroidism: TSH <0.005 mIU/L (reference range 0.27–4.2 mIU/L), free triiodothyronine (FT3) 31.7 pmol/L (reference range 3.1–6.8 pmol/L), free thyroxine (FT4) 56.9 pmol/L (reference range 12.0–22.0 pmol/L), serum thyroglobulin (TG) concentration >5000 μg/L (reference range <77 μg/L), anti-TSH-R 82.2 U/L (reference range <1.8 U/L). Before referring to our institute, a CT examination of the neck and chest with contrast media showed a thyroid tumor, metastatic lymph node on the left side of the neck and revealed metastases in the lungs, mediastinum, and in the 8^th^ right rib measuring 20 × 5.6 × 4.5 cm, in the left acetabulum measuring 9 × 9 × 3 cm and parietooccipitally in the skull measuring 5 × 4 × 2 cm. Estimated total volume of tumor tissue was at least 515 mL (primary tumor 113 mL, 8th rib 263 mL, left hip 127 mL, and skull metastasis 21 mL).

Fine-needle aspiration biopsy and cytological investigation revealed metastases of HCTC in the skull and in the 8^th^ right rib. The multidisciplinary team recommended establishment of euthyroid state and external-beam irradiation (EBRT) of the left hip because of threatening fracture. The region of the left hip had been irradiated with 10 × 3 Gy and doxorubicin 20 mg once weekly was given concomitantly. Hyperthyroidism was controlled with thiamazole in the following two weeks. After this treatment, the primary tumor decreased in size by about 15%, became movable and skeletal pain diminished in intensity.

A total thyroidectomy and R1 resection of the thyroid tumor was performed in December 2017. Histological examination revealed a widely invasive HCTC in the left lobe measuring 9 cm in diameter with microscopic penetration of the thyroid capsule and focal growth into the dorsal surgical margin (tumor, nodes, metastases stage T3N1M1).

**Figure 1:**
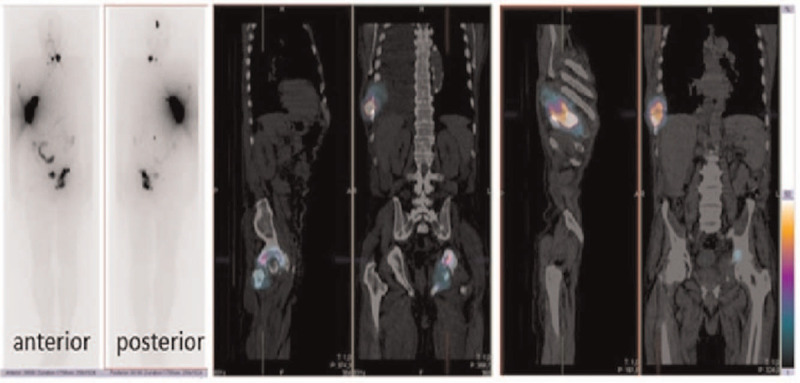
SPECT/CT with iodine-123 (January 2018) after total thyroidectomy showed a large pathologic accumulation in the 8th right rib and additional pathologic accumulations in the thyroid remnant, on the left side of the neck, in the left acetabulum, in the skull and in the right humerus.

**Figure 2:**
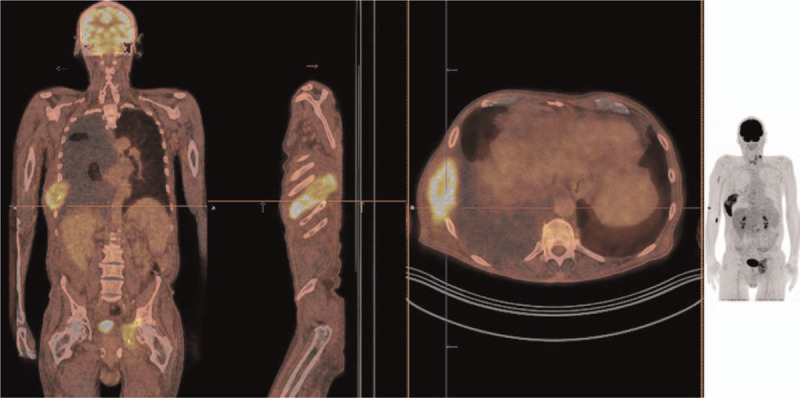
18F-PET/CT After thyroidectomy and before the 8th rib resection (February 2018).

After thyroidectomy, the patient stopped thiamazole therapy and started treatment with l-thyroxine. However, T3 hyperthyroidism persisted and the patient was instructed to stop taking L-thyroxine. In January 2018, SPECT/CT with iodine-123 showed a large pathologic accumulation in the 8^th^ right rib and additional pathologic accumulations on the left side of the neck, in the left acetabulum, in the skull and in the right humerus (Fig. 1). Furthermore, investigation with ^18^F-FDG-PET/CT (Fig. 2) showed an extensive metastasis with increased activity in the 8^th^ right rib with extensive pleural effusion on the right side, activity in the lymph node metastasis on the left side of the neck, an extensive metastasis of moderate to high activity in the left acetabulum, metastases of low activity in the skull, and the right humerus and small nodular changes in the lungs. Compared to iodine-123 scintigraphy, no additional accumulations of radiopharmaceutical were seen. A cytological examination showed no malignant cells in pleural fluid.

In February 2018, euthyroidism was established after 2 weeks’ therapy with thiamazole and the 8^th^ right rib was resected (R1 resection). Histologically, the rib tumor was a metastasis of HCTC with a diameter of 20 cm. The tumor destroyed the rib, was necrotic, and grew into the surrounding soft tissues. After the rib resection, the patient stopped therapy with thiamazole and l-thyroxine suppressive therapy was reintroduced.

In March 2018, 4 weeks after the rib resection and 3 months after CT investigation with contrast media, an ablation/therapy with 146 mCi of radioactive iodine-131 (RAI) was performed. RAI accumulated in the skull, thyroid remnant, and lymph node in the left side of the neck. Furthermore, in the left acetabulum uptake was extensive, whereas it was moderate in the diaphysis of the right humerus and numerous metastases in the lungs.

For the next 2 months, the patient was feeling good and was without pain or dyspnea. The skull metastasis diminished in size and measured only 1 × 1 cm. Therapy with bisphosphonates was initiated after another 2 months.

**Figure 3:**
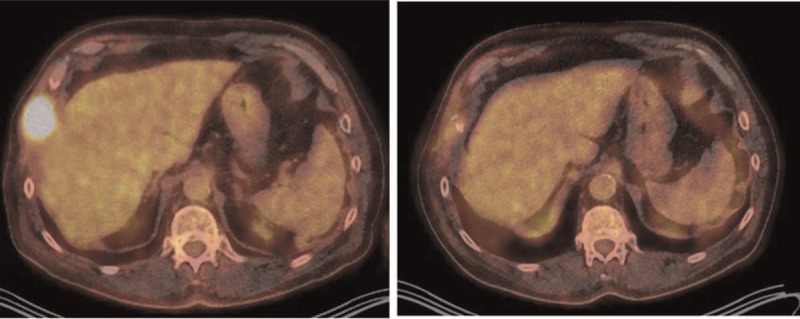
18F-PET/CT: Left image—A recurrence in the stump of the 8th rib in September 2018; Right image—After the 2nd radioiodine therapy and external beam radiotherapy, a regression of the recurrence in the stump of the 8th rib was seen in February 2019. Unfortunately, this regression was only temporary, so treatment with sorafenib was initiated in April 2019.

In September 2018, ^18^F-FDG-PET/CT (Fig. 3) showed metabolic progression of metastases in the skull and a recurrence of the metabolic activity in the stump of the 8^th^ right rib and in the 7^th^ right rib. However, a partial metabolic effect of RAI therapy in the neck lymph node metastasis, partial metabolic regression in the left acetabulum and buttock, and right humerus was also seen.

In October 2018, SPECT/CT with iodine-123 during hormonal withdrawal showed less pronounced uptake in the skull, neck lymph node metastasis and left acetabulum compared to the previous scan with ioiodine-123. However, there was a new accumulation in the stump of the 8^th^ rib and in the lung. Therapy with 174 mCi RAI during hormonal withdrawal (TSH 28.3 mU/L) showed accumulations of the same metastases as with iodine-123 scan. In November 2018, the stump of the 8^th^ right rib was treated with EBRT (12 × 3 Gy) because of metabolic progression of the disease. Molecular analysis of the primary tumor was performed and showed a mutated NRAS c.182A> G p. (Gln61Arg) gene and a mutated AKT1 c-49G> A p. (*Glu17Lys*) gene. However, in the *KRAS* gene, as well as in the *BRAF* gene, mutations were not detected.

In February 2019, ^18^F-FDG-PET/CT (Fig. 3) showed metabolic progression in the skull, the right humerus and the 7^th^ right rib, whereas metabolic stagnation was seen in the metastasis of the left acetabulum and regression in the stump of the 8^th^ rib and the metastasis in the neck lymph node.

In March 2019, another course of therapy with 195 mCi RAI during hormonal withdrawal was given. Post-therapy I-131 scintigraphy showed an uptake in the neck lymph node metastasis, in the thyroid bed, in the lungs, 7th right rib and left acetabulum. The uptake was minimal in the skull and in the diaphysis of the right humerus. Furthermore, the progression of the skull, right humerus, and 7^th^ right rib metastases and partial regression of the metastasis in the neck lymph node, lungs and left acetabulum was seen by ^18^F-FDG PET/CT in March 2019.

Following this progression of the disease and after receiving a total of 515 mCI of radioiodine, sorafenib with a dose 2 × 400 mg was initiated in April 2019. In May and September 2019, the patient reported less skeletal pain than before. Side effects were mild. Laboratory investigations showed a decrease of Tg concentration <5000 μg/L and a mild hepatopathy. In September 2019, ^18^F-FDG-PET/CT showed mixed response to therapy: a progression in the 7^th^ right rib and the stump of the 8^th^ rib, stagnation in the left acetabulum and lungs, and regression in the skull, neck lymph node, and right humerus.

**Figure 4:**
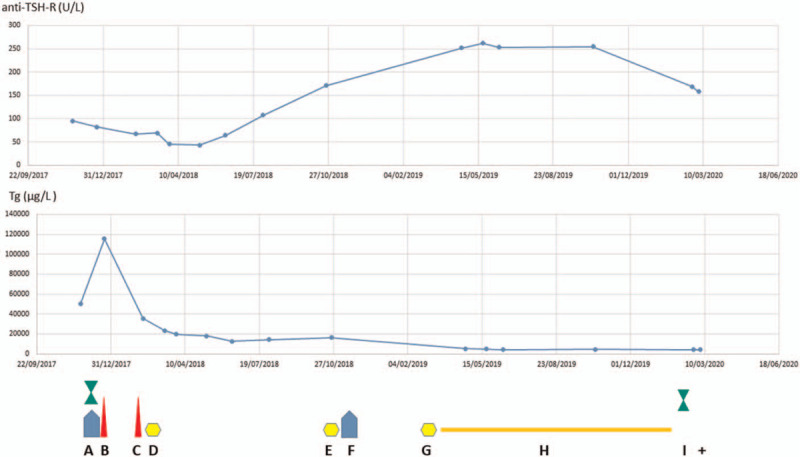
Treatment and concentration of thyroglobulin (Tg) and antibodies against TSH-R (anti-TSH-R). (A) External beam irradiation (EBRT) and concurrent chemotherapy with doxorubicin of the left hip; (B) total thyroidectomy; (C) resection of the 8th right rib; (D) ablation/therapy with radioiodine (RAI); (E) RAI therapy; (F) EBRT of the stump of the 8th right hip; (G) RAI therapy; (H) targeted therapy with sorafenib; I: chemotherapy with cyclophosphamide, doxorubicin and vincristine; +: patient died. Legend of symbol colours: blue: EBRT, red: surgery, yellow: RAI therapy; orange: targeted therapy; green: chemotherapy.

In October 2019, laboratory investigations showed optimal TSH suppression, Tg 4552 μg/L and anti-TSH-R = 255 U/L (Fig. 4).

In December 2019, severe fatigue and weight loss due to altered taste and decreased appetite were reported. The patient had occasional arterial hypertension, so the sorafenib dose was reduced to 200 and 400 mg/day.

In February 2020, the patient was admitted to hospital because of severe dyspnea due to pleural effusion on the right side. Cytological investigation revealed malignant cells of HCTC. Thoracentesis and pleurodesis were not beneficial. In March 2020, a CT scan of the chest and abdomen showed marked progression of the pleural effusion on the right side, septic or metastatic embolisms in the right side of the lung and a peritoneal tumor measuring 18 × 8 × 5 cm. Cytology confirmed a metastasis of HCTC.

Due to rapid progression in terms of visceral crisis, we started chemotherapy with cyclophosphamide, doxorubicin, and vincristine in a reduced dose. There was no effect of chemotherapy on the tumor. Due to the severe deterioration of his general condition and uncontrolled symptoms, the patient was transferred to the acute palliative care department. The leading symptoms were severe dyspnea, restlessness, and pain. After the introduction of analgesia with an elastomeric pump, the pain ceased. After a family meeting with the patient and his relatives, we decided on supportive palliative treatment. The patient died due to cardiorespiratory failure in March 2020; thus, he survived for 28 months from diagnosis.

## 3 Discussion

A recent meta-analysis demonstrated an increased risk of distant metastasis at the time of cancer diagnosis in patients with differentiated thyroid cancer and GD in comparison to those without GD.^[11]^ Furthermore, Pellegriti et al^[12]^ found increased disease-specific mortality in patients with GD in comparison with matched euthyroid patients with thyroid cancer. Our case report confirms the data from the literature. The patient survived for only 28 months from diagnosis, whereas 10-year disease-specific survival for all patients with metastatic HCTC, pulmonary metastases, and bone metastases was 60%, 60%, and 68%, respectively.^[10]^ The shorter survival of our patient might have been related to the stimulating action of anti-TSH-R and very advanced disease at diagnosis.

In our patient with metastatic HCTC and GD, T3 hyperthyroidism at initial evaluation was most likely the result of the stimulating activity of anti-TSH-R. In GD, relatively more T3 than T4 is produced and the ratio between FT4 and FT3 was shown to be significantly lower than in other types of hyperthyroidism.^[13]^ Through stimulation of TSH-R, anti-TSH-R induced the expression of sodium iodide symporter, a key plasma membrane transport protein on the basolateral membrane of thyrocyte that enables active transport and thyroid uptake of iodide as well as radioactive iodine-123 or iodine-131.^[3,14]^ Unfortunately, before admittance of the patient to our institution, a CT investigation with contrast media was performed during a diagnostic procedure. The CT contrast media probably provoked hyperthyroidism. RAI extensively accumulated in the 8^th^ right rib, skull, left acetabulum, thyroid remnant, lymph node in the left side of the neck and moderately in the diaphysis of the right humerus and numerous metastases in the lungs. Accumulation of RAI in primary HCTC and metastatic sites in our patient concurs with our earlier observation that RAI accumulated in metastatic HCTC.^[9,15]^ In our case, the accumulation was most likely additionally increased as a result of the anti-TSH-R stimulatory action on thyroid uptake of iodide. Of course, in our patient, the effectiveness of RAI therapy was limited due to the extremely large volume of metastases. It is well known that the efficacy of RAI therapy is related to the mean radiation dose delivered to neoplastic foci.^[16,17]^

Multimodal treatment of metastatic thyroid carcinoma may be an effective approach in HCHT.^[9]^ Only recently, neoadjuvant therapy with tyrosine kinase inhibitor therapy has been reported to be effective in reducing the primary tumor size before total thyroidectomy.^[18]^ Very recently, sorafenib^[19]^ and lenvatinib^[18]^ were reported as neoadjuvant therapy to reduce tumors sufficiently to enable thyroidectomy. In our patient, the left hip metastasis was initially treated by EBRT and concomitant chemotherapy with doxorubicin. After chemotherapy, the primary tumor decreased in size. Neoadjuvant chemotherapy may be effective in patients with HCTC in 43% in whom tumor size decreased by >50%.^[20]^ The other factor that probably caused a reduction of the primary tumor size was decreased blood perfusion of thyroid tissue after therapy with thiamazole.

Concomitant external beam radiotherapy and doxorubicin chemotherapy, followed by RAI therapy, prevented the growth of a large metastasis in the left hip in our patient. Effective treatment with concomitant EBRT and chemotherapy was also reported by Beckhman et al from Memorial Sloan Kettering Cancer Center in New York.^[21]^ They reported that the use of intensity-modulated radiation therapy with concurrent chemotherapy with doxorubicin is a safe and effective treatment to achieve local control in patients with unresectable or incompletely resected nonanaplastic, nonmedullary thyroid cancer. Their opinion is that concurrent doxorubicin should be considered in these patients to improve local control and survival.^[21]^

A large metastasis in the 8^th^ right rib presented an unresolved problem in our patient. Persistent T3 hyperthyroidism, most likely caused by TSH-R-stimulated T3 production in large metastasis in the 8th right rib, was eliminated by rib resection. Unfortunately, the multidisciplinary team did not decide to recommend immediate postoperative EBRT and concomitant chemotherapy, even though the resection was only macroscopically radical. They expected that treatment with RAI would be effective because of the high uptake of iodine in this metastasis and the remnant of the tumor after rib resection was small. Additionally, when a recurrence in the stump of the 8^th^ right rib was treated by EBRT, concomitant chemotherapy was not applied because the radiotherapist did not choose to use it. Nonconcomitant treatment of this region with RAI, followed by EBRT at the time of large tumor recurrence, was not effective.

During our treatment, we were not able to control the level of anti-TSH-R. Initially, the concentration of anti-TSH-R temporarily decreased, most likely as a result of treatment with thiamazole and subsequent resection of a large volume of tumor burden, including the thyroid gland and the 8^th^ right rib. Three months after the ablation of the remaining thyroid tissue with RAI therapy, the concentration of anti-TSH-R significantly increased. A similar increase of anti-TSH-R is frequently observed in RAI-treated GD patients and presumably, it is caused by the release of thyroid antigens during RAI-induced thyroid destruction.^[22]^ However, during recurrence of the 8^th^ right rib metastasis and after the second and third RAI application, the concentration of anti-TSH-R increased further, most likely as a reflection of tumor burden enlargement and the release of thyroid antigens after RAI therapy.

In differentiated thyroid cancer, targeted therapy should be started when the disease becomes radioresistant or the cumulative dose of radioiodine is exceeded. The most appropriate timing to start targeted therapy is contradictory because of the tumor escape phenomenon when disease progression becomes very aggressive and rapid.^[18,23]^ Ahmaddy et al reported that tumor response assessment by ^18^F-FDG-PET outperforms morphological response assessment in CT in patients with advanced, radioiodine-refractory differentiated thyroid cancer, and seems to be associated with clinical outcome.^[24]^ Recent European Society of Medical Oncology guidelines recommend targeted therapy with sorafenib or lenvatinib in patients with progressive radioiodine-refractory disease after careful assessment of the extent of the disease and discussion with the patient.^[25]^ Some authors advocate the use of tyrosine kinase inhibitors not only when a progression of the metastatic lesions is documented, but also when the tumor burden is very advanced, provided their radioiodine refractoriness.^[23]^ Our patient received a cumulative dose of 515 mCi of radioiodine and after the disease proved to be radioiodine-refractory, treatment with sorafenib was started. Although a metabolic progression with PET-CT was obvious, only a proven structural progression or radioactive iodine-refractory state was needed before treatment with sorafenib was initiated. The duration of sorafenib treatment was 10 months. This is in concordance with reported results in the multicenter, randomized, double-blind, placebo-controlled, phase 3 DECISION trial, which reported median duration of treatment of 10.6 months.^[26]^ There is a question whether immediate second-line targeted therapy would slow down rapid progression of disease after the termination of sorafenib treatment. However, an infection and the poor general condition of patient that complicated pleurodesis precluded the introduction of a possible second-line targeted therapy, which would be sunitinib, which was the one available at that time in Slovenia. There are no data to indicate that any of the patients with concomitant GD were included in the DECISION or SELECT trials.

In the present case report, we describe the progressive course of the disease and management of a patient with Graves disease and metastatic HCTC who was treated by external beam irradiation with concomitant chemotherapy, surgery, radioiodine therapy, and targeted therapy. Our study has several limitations. The first limitation of our study is the retrospective and nonrandomized nature. Furthermore, from our case report it is not possible to find out which is optimal multimodal treatment in patients Graves’ disease and metastatic hormonal-active HCTC. However, to our knowledge there are no data in the literature about the effectiveness of external beam irradiation with concomitant chemotherapy, surgery, radioiodine therapy, and targeted therapy in such patients.

## 4 Conclusions

Simultaneous hormone-active HCTC and GD is extremely rare and prognosis is dismal. Our case report is interesting because it describes the treatment of an individual with this rare entity by combined multimodal treatment, which also included sorafenib. Our view is that multimodal approach should be used in patients with hormone-active metastatic Hürthle cell thyroid carcinoma and Graves disease.

## Author contributions

**Conceptualization:** Nikola Besic, Katja Zaletel.

**Data curation:** Nikola Besic, Barbara Vidergar-Kralj, Katja Zaletel.

**Figures:** Barbara Vidergar- Kralj, Katja Zaletel.

**Funding acquisition:** Nikola Besic, Katja Zaletel.

**Visualization:** Barbara Vidergar-Kralj, Katja Zaletel.

**Writing – original draft:** Nikola Besic.

**Writing – review & editing:** Nikola Besic, Barbara Vidergar-Kralj, Katja Zaletel, Cvetka Grasic-Kuhar.
